# A Personalized QoS Prediction Approach for CPS Service Recommendation Based on Reputation and Location-Aware Collaborative Filtering

**DOI:** 10.3390/s18051556

**Published:** 2018-05-14

**Authors:** Li Kuang, Long Yu, Lan Huang, Yin Wang, Pengju Ma, Chuanbin Li, Yujia Zhu

**Affiliations:** School of Software, Central South University, Changsha 410075, China; yl4548@csu.edu.cn (L.Y.); huanglan@csu.edu.cn (L.H.); 164712103@csu.edu.cn (Y.W.); mapengju@csu.edu.cn (P.M.); 164712139@csu.edu.cn (C.L.); 164711017@csu.edu.cn (Y.Z.)

**Keywords:** cyber-physical systems, QoS prediction, collaborative filtering, data sparsity, user reputation, geographical neighbors

## Abstract

With the rapid development of cyber-physical systems (CPS), building cyber-physical systems with high quality of service (QoS) has become an urgent requirement in both academia and industry. During the procedure of building Cyber-physical systems, it has been found that a large number of functionally equivalent services exist, so it becomes an urgent task to recommend suitable services from the large number of services available in CPS. However, since it is time-consuming, and even impractical, for a single user to invoke all of the services in CPS to experience their QoS, a robust QoS prediction method is needed to predict unknown QoS values. A commonly used method in QoS prediction is collaborative filtering, however, it is hard to deal with the data sparsity and cold start problem, and meanwhile most of the existing methods ignore the data credibility issue. Thence, in order to solve both of these challenging problems, in this paper, we design a framework of QoS prediction for CPS services, and propose a personalized QoS prediction approach based on reputation and location-aware collaborative filtering. Our approach first calculates the reputation of users by using the Dirichlet probability distribution, so as to identify untrusted users and process their unreliable data, and then it digs out the geographic neighborhood in three levels to improve the similarity calculation of users and services. Finally, the data from geographical neighbors of users and services are fused to predict the unknown QoS values. The experiments using real datasets show that our proposed approach outperforms other existing methods in terms of accuracy, efficiency, and robustness.

## 1. Introduction

Technical advances in ubiquitous sensing, embedded computing, and wireless communication have led to a new generation of engineered systems called cyber-physical systems (CPS) [1]. CPS has changed the way that we interact with the physical world. It provides a smart infrastructure to connect abstract computational artifacts with the physical world, and breaks the boundary between the cyber world and the physical world [2]. CPS integrates multiple techniques, including distributed computing, communication, and automatic control, to support a variety of intelligent services and applications in many fields, such as transportation, healthcare, entertainment, and city infrastructure [3,4,5]. In CPS, wireless sensors or the Internet of Things collect information from web networks and various mobile devices, thus generating a large amount of data [6]. CPS provide a variety of services for our life by collecting a large amount of data. However, due to a large increase in CPS services, it is difficult for users to select high-quality CPS services, so it becomes an urgent task to effectively recommend suitable candidate services for different users. 

There are two kinds of attributes for CPS services, namely, functional and non-functional attributes. With the increase of CPS services, there are more and more competitive services with similar functionality, and non-functional attributes become a key factor in service selection, since they can differentiate the performance of candidate CPS services with the same or similar functionality. Quality of service (QoS) becomes a hot spot in current service computing and distributed computing research [7]. In the real world, the values of some user-dependent QoS properties (for example, response time, throughput, and so forth) may vary largely because of different users, physical positions, network conditions (4G, 5G, Wi-Fi, and so forth), and other objective factors [8]. It is impractical for a single user to simply attempt all candidate CPS services to obtain sufficient QoS data for evaluation, therefore, it is important to predict user-dependent QoS values for effective CPS services recommendation.

There is no doubt that collaborative filtering (CF) has recently been widely used in QoS prediction methods, especially neighborhood CF [9,10,11,12,13,14,15,16,17,18,19,20,21,22,23]. The neighborhood CF-based approach typically consists of two steps: (1) Finding similar users (or services) and mining their similarity; and (2) calculating unknown QoS values based on existing historical data of similar users (or services). However, there are two main problems in existing approaches: (1) The data are usually very sparse when calculating the similarity of users (or services). When the QoS matrix is extremely sparse, the QoS values observed by users may be very few or even null. In this case, it is difficult to calculate user (or service) similarity accurately, and data sparseness will reduce the accuracy of predicting QoS values. (2) It is usually assumed that all users are honest and they will provide reliable QoS data. However, in reality, some users may submit random or unchanging values, or some users (for example, service providers) may provide good QoS values for their own services and provide poor values to their competitors [13]. Most existing approaches fail to identify and pre-process the unreliable QoS data before QoS prediction. Therefore, an effective QoS prediction approach, which can not only deal with data contributed by untrusted users, but also mitigate the impact of data sparseness and the cold start problem [24], and finally achieve high prediction accuracy, is required for a robust CPS service recommender system.

In order to solve the two problems, in this paper, we propose a personalized QoS prediction approach named GURAP (Geographic location and User Reputation Aware Prediction) based on reputation and location-aware collaborative filtering for CPS services. Firstly, in order to deal with unreliable data contributed by untrusted users, we propose to calculate the reputation of users based on the collected QoS data and the Dirichlet probability distribution, then we can identify the untrusted users based on a reputation threshold and fix the QoS contributions from the untrusted users. Secondly, in order to mitigate the impact of data sparseness and the cold start problem, we propose to find out the user and service neighbors from the perspective of geographical location on three levels, and then improve the similarity calculation of users and services. Thirdly, in order to achieve high prediction accuracy, we propose an improved CF method to predict missing QoS values based on the QoS records of similar users and similar services. The extensive experiments conducted in Section 5 demonstrate the validity and robustness of our approach.

The rest of this paper is organized as follows. In Section 2, we review the related work on CPS services QoS prediction. In Section 3, we introduce our proposed QoS prediction framework for CPS services. The details of our QoS prediction approach are presented in Section 4. Then the proposed approach is evaluated with an extensive experiment on a public dataset WSDream in Section 5. Finally, conclusions and future work are given in Section 6.

## 2. Related Work

Service oriented computing (SOA) has been widely studied in service composition [25,26], service selection [27,28], service recommendation [29], and service discovery [30]. These studies have been proved valuable in many areas, such as distributed computing, cloud computing, Internet of Things, and networked physical systems. As the number of CPS services continues to increase, it becomes more and more difficult for users to efficiently find suitable services from a large number of services. Therefore, most recommender systems use QoS-based methods to recommend high-quality services to users. However, as services typically run on dynamic networks, the values of some QoS attributes (for example, response time, throughput, and so forth) are affected by the context of different users, such as geographical location and network conditions. Therefore, an accurate and personalized QoS prediction method is crucial for the successful implementation of a QoS-based service recommender system.

At present, QoS prediction approaches for services can mainly be classified into three types: Time series [31], context-based [32], and CF-based methods. Furthermore, the CF-based approaches can be mainly divided into two kinds: Neighborhood-based (memory) CF and model-based CF. The neighborhood-based CF approaches predict the missing QoS values by utilizing the observed QoS values of similar users or similar services. Shao et al. [11] first used collaborative filtering to realize QoS prediction, and they proposed a user-based CF method to predict QoS values. Zheng et al. [33] proposed a hybrid CF approach (UIPCC) of predicting the QoS values of services, and conducted a large-scale distributed QoS assessment of real-world services. Chen et al. [34,35] proposed a sort-oriented approach that combines project-based collaborative filtering with potential factor models to address service ranking. Hu et al. [31] proposed a QoS prediction approach considering both the temporal dynamics of QoS attributes and the personalized factors of service users. The approach seamlessly combines the CF method with the improved time series method and uses the Kalman filter to compensate for the shortcomings of the ARIMA model. These neighborhood-based CF approaches indicate that sufficient QoS entries are required for QoS prediction with normal performance.

The model-based CF predicts the missing entries primarily by using the observed data to train a predefined factor model. Matrix factorization (MF) is a typical model-based approach in collaborative filtering that has been extended in many applications. Luo et al. [21] established a collaborative filtering model based on matrix factorization for QoS prediction under non-negative condition constraints. They dealt with non-negative-bound QoS matrices through non-negative latent factor models and introduced Tikhonov regularization to obtain regularized non-negative latent factor models. Zhang et al. [22] proposed a service QoS prediction framework called WSPred, which mainly adopted the tensor factorization technique to fit the factor model of user–service-temporal tensor, and used factorization to combine user-specific, service-specific, and temporal feature matrices to predict missing values. Xie et al. [23] considered hidden correlation (asymmetric correlation) between users and services, and proposed an asymmetric correlation regularized matrix decomposition (MD) framework for service recommendation. 

Most of the traditional approaches directly use the historical QoS values provided by users to predict the unknown QoS values, without considering whether the QoS values are reliable or not. Therefore, in order to improve the accuracy of QoS prediction, some researchers propose to improve the traditional approaches based on user reputation. Qiu et al. [14] proposed a reputation-aware prediction approach, which calculated users’ reputation based on the QoS value contributed by the user, and then filtered the users with low reputation and used the purified QoS data to predict the missing value. However, the exclusion of the QoS data contributed by low reputation users causes the data matrix to intensify in sparseness. Therefore, Xu et al. [15] proposed a CF method based on reputation matrix decomposition, which directly integrates user reputation into the factor model learning process. However, the reputation calculation depends on many parameters, and improper parameter settings may result in poor performance. In order to overcome the limitation, Wu et al. [16] proposed a credibility-aware QoS prediction (CAP) method, which employs two-stage K-means clustering to identify untrustworthy users. However, this approach only uses credible similar users to predict the values, without considering the valuable information of the services. Thus, Su et al. [17] proposed a trust-aware prediction (TAP) method for the reliable personalized QoS prediction. They use the K-means clustering algorithm and Beta-based probability distribution method to calculate users’ reputation degrees first, and the degrees are used as weights of the original QoS matrix, and finally the missing QoS values are predicted based on the weighted QoS matrix. The above two approaches mainly solve the problem of untrustworthy users on the accuracy of QoS prediction, but they do not solve the data sparseness and cold-start problems.

In order to alleviate the problem of highly sparse data in the real world, many researchers have proposed methods to counteract this. In reference [36], Chen et al. proposed a hybrid QoS prediction approach, which uses a hierarchical clustering to integrate services with the same physical environment, rather than a separate service, to excavate the user’s similarity. Recently, some researchers have begun to realize the importance of geographic location information on QoS prediction. For example, Tang et al. [9] represented the location of users and services as three-layers (IPu, ASNu, CountryIDu) and used Pearson correlation coefficient (PCC) to find similar users or similar services at different geographic regions. This approach not only reduces the search space, but also alleviates the problem of sparse matrix to a certain extent. In reference [18], Chen et al. validated that the QoS value of the service that was invoked by the user was significantly influenced by the geographic neighborhood of the service, based on the empirical data of the real world QoS data set. They use hierarchical neighborhood clustering to classify the services into three-tiered geographical regions based on the implicit geographic information of the service. However, there is no guarantee in the national geographic region that there will be enough geographic neighbors, and it also did not consider the impact of users’ geographic information on the projections. These approaches alleviate the problem of sparseness and cold start of the matrix to a certain extent, and also improve the accuracy of QoS prediction. However, these approaches are based on the assumption that the user is trusted and the QoS value provided is reliable. In the real world, the user is not necessarily credible, and the QoS value provided by the untrustworthy user will reduce the QoS prediction accuracy.

Through previous research work, we can find that most of the approaches do not consider both of the problems on reliable QoS preprocessing and data sparseness in QoS prediction. Hence, we firstly propose the GURAP approach to solve the two challenges simultaneously. Our approach can be divided into three steps. In the first step, we identify the untrusted users and amend the QoS values contributed by them with the average value of the reliable QoS interval for each CPS service. In the second step, we classify users/services according to geographic locations in three levels, then calculate the relevance degrees between every two levels of geographic neighbors to improve the calculation of Pearson correlation, and then select the top-K similar geographical neighbors according to the similarity of users/services. In the third step, an improved collaborative filtering method is proposed to calculate the missing QoS values based on the reliable QoS records from the selected top-K similar users/services.

The principal contributions of this paper can be summarized as follows:

- We propose an improved method for calculating users’ reputation, based on the approach in reference [17]. Different to the probability model and the treatment of data from the identified unreliable users, our method uses statistical interval partitioning, Gaussian normal distribution, and Dirichlet probability distribution to calculate users’ reputation. Next, the untrusted users are identified and their unreliable QoS data are fixed by the mean value of reliable users. This ensures the credibility of user data and avoids exacerbating the sparseness of QoS data due to the direct deletion of unreliable data.
- In order to identify similar users/services and alleviate data sparseness, we propose a geographic proximity recognition algorithm based on the locations of users/services. The algorithm fully exploits the geographic relevance of users/services and improves the Pearson correlation formula to identify the neighbors of users/services in geographic location.
- We propose a QoS prediction approach (GURAP) based on reputation and geographic location-aware collaborative filtering. The approach can predict unknown QoS values, based on reliable QoS data from users and services with geographic proximity. We simultaneously consider the impact of data reliability and data sparseness on the prediction accuracy for the first time. We conduct extensive experiments with real world data sets to study the prediction accuracy and robustness of our approach by comparing with classical approaches.

## 3. QoS Prediction Framework for CSP Services

**Figure 1:**
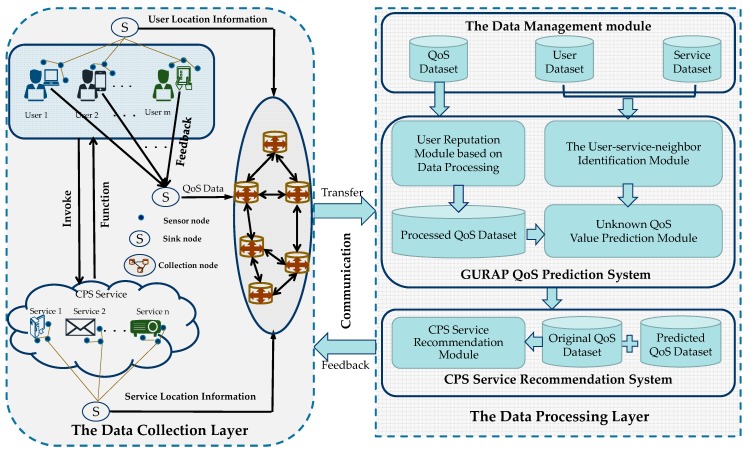
The quality of service (QoS) prediction framework for cyber-physical system (CPS) services.

Recently, researchers proposed frameworks such as WSRec [33], EMF [37], and HMF [20] to collect QoS data contributed by users and use them to predict unknown QoS values. However, these prediction frameworks ignore the reliability of the collected data. In fact, QoS values may be unreliable since they can be provided by untrusted users. Therefore, some researchers proposed prediction frameworks such as RAP [14] and TAP [16] to calculate users’ reputation to identify untrusted users, so as to ensure that the used data are reliable. However, these frameworks ignore the effect of data sparsity on the prediction as the QoS prediction accuracy would be reduced when the data are extremely sparse. To meet the major challenges, we propose a highly reliable QoS prediction framework, based on reputation and location-aware collaborative filtering, as shown in Figure 1.

In Figure 1, in the QoS prediction framework for CPS services, the data collection layer submits the QoS data of all users invoking CPS services, and the geographic location information of users and services, to the data processing layer. Then the data processing layer predicts the unknown QoS values for trusted users, based on the collected data. Finally, the QoS prediction framework transmits the predicted QoS information to the CPS service recommendation system to recommend the appropriate services for the user. Specific steps are as follows.

1. First, in the data collection layer, the QoS records of users’ invocation of CPS services, as well as the geographic location information of users and CPS services, are collected and transmitted to the collection node through the internet. The collection node then transfers the collected information to the data management module in the data processing layer.
2. The data management module is in charge of information extraction, transformation, and load from multiple sources, and the collected data are classified to three kinds, namely the QoS data, users’ locations, and services’ locations.
3. In the user reputation recognition module, the calculation unit performs pre-processing on the QoS data matrix, identifies the untrusted users, and processes the unreliable QoS data to form a new QoS matrix.
4. In the user-service-similar-neighbor identification module of the data processing layer, the module computing unit firstly mines the correlation between the geographical locations of the users or services through calculation, and then identifies the similar geographical neighbors of users/services, according to the new QoS matrix.
5. In the unknown QoS prediction module of the data processing layer, the module predicts the unknown QoS values of the trusted user based on the reliable QoS records from the selected top-K similar users/services.
6. In the last step, the predicted unknown QoS value is transmitted to the CPS service recommendation system to select a high-quality CPS service for the user. Afterward, the process starts from step 1 again, thus forming a CPS service prediction recommendation system for computing, communication, and control.

## 4. QoS Prediction Approach Based on Reputation and Location-Aware Collaborative Filtering

### 4.1. The Process of GURAP

**Figure 2:**
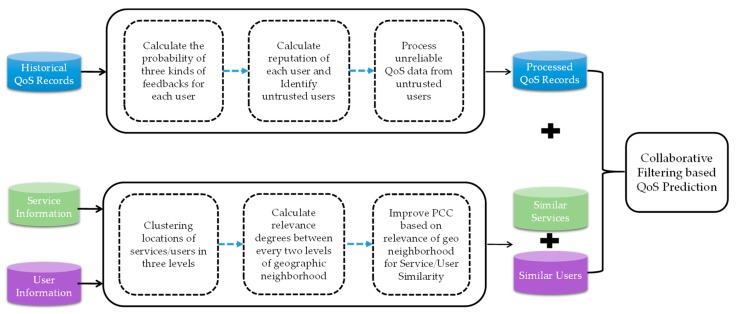
Process of QoS prediction, based on geography and reputation-aware.

In this section, we will introduce the personalized QoS prediction approach, GURAP, based on geographic location and reputation-aware collaborative filtering for CPS services. As shown in Figure 2, the main process of GURAP consists of three parts: The user reputation-based calculation part (CURA), the geographic-based user (service) similarity neighbor recognition part (GUIPCC), and the unknown QoS value prediction part, based on users and services. The details of each process are described in Section 4.2, Section 4.3 and Section 4.4 respectively.

### 4.2. User Reputation-Based Calculation

QoS is a collection of properties that describe the non-functional nature of CPS services, where each property represents one aspect regarding CPS services [38]. According to the characteristics of QoS attributes, we can classify the QoS attributes into two categories: One is collected and analyzed on the service side, and the values are the same for different users (for example, cost, availability, popularity, and so forth); the other kind is the attributes depending on users, and the values vary among different users due to their different locations, network environment, and so on (for example, response time, throughput, and so forth) [39]. Please note that we only deal with the prediction of user-dependent QoS metrics in this paper. Since the factors associated with each user are not the same, different users have different QoS values for the invocation of a same CPS service. However, under normal circumstances, the QoS values observed by most users for each service should fall within the normal range, and an observation highly deviating from the normal value is unlikely to happen [17]. Therefore, if a user always submits QoS feedback highly deviating from the majority, this user may be untrustworthy. Based on this principle, we can evaluate the probability whether the user is trustworthy based on the information of user’s feedback on previous invocation records. The following is the detailed introduction of CURA.

We first assume that there is a set of m users U = {$u_{1},u_{2},...,u_{m}$} in the system, a set of n services S = {$s_{1},s_{2},...,s_{n}$}, and the user service matrix is an *m* × *n* matrix R. Each entry in the matrix $r_{i,j}$ (*i* ≤ *m*, *j* ≤ *n*) represents the value of a certain user-side QoS attribute (for example, response time) of the CPS service $s_{j}$ observed by the user $u_{i}$. If user $u_{i}$ has not invoked the CPS service $s_{j}$ before, $r_{i,j}$ is equal to 0. The QoS matrix is as shown in Table 1. In practice, it is not possible for a user to invoke all of the services to obtain QoS values, so in the real world many QoS values are unknown.

#### 4.2.1. Calculating User’s Feedback Vector

In order to get a feedback vector of each user, we divide the task into three steps: Normalizing the QoS data, determining the reliable user cluster, and calculating the user feedback vector.

(1) Normalizing QoS data

Because the range of QoS values for all users invoking each service is different, in order to divide the statistical interval more conveniently, the QoS value $r_{i,j}$ of each service needs to be normalized. We normalize the QoS data using a linear function normalization method shown in Equation (1):

(1) $$nr_{i,j}=\frac{r_{i,j}-r_{j}^{\min}}{r_{j}^{\max}-r_{j}^{\min}}$$

where $nr_{i,j}$ denotes the normalized value of $r_{i,j}$, $r_{j}^{\min}$ is the smallest QoS value of service *j*, and $r_{j}^{\max}$ is the largest QoS value of service *j*. When the service QoS value invoked by the user is 0 (missing value), the value is 0 after normalization.

(2) Determining reliable user cluster

In order to determine reliable user clusters, we separate the QoS values of all users, for each service, in the normalized QoS matrix to UK statistical intervals, according to the statistical interval division method and the normalized QoS value. For example, the QoS data in [0, 1] intervals can be evenly divided into 11 intervals of (0, 0.1], (0.1, 0.2], ..., (0.9, 1], and [0, 0]. For all users, the users who invoke services are similar [16,36]: Trustworthy users always take the major part of all users, and their QoS values are in the normal range, so the intervals that contain the most users can be considered reliable. The user cluster in the reliable interval on service *j* is defined as:

(2) $$U_{j}^{\max}=\{u|u\in C_{j}^{t},t=\underset{k}{\arg\max}\left|C_{j}^{k}\right|,0\leq k\leq (UK-1)\}$$

where $U_{j}^{\max}$ denotes the set of users in a reliable interval on service *j*, $\left|C_{j}^{k}\right|$ denotes the number of users in the *k*th interval, and *t* denotes the index of the interval with the largest number of users. When determining the reliable interval, the number of users in the [0, 0] interval is not counted.

(3) User’s feedback vector

When the QoS value of the service invoked by the user is 0, the user has no feedback on the service. Since a reliable user set $U_{j}^{\max}$ reflects the result of most users after invoking the service, QoS values submitted by the user in a reliable interval would be closer to the normal value of service *j*. As mentioned earlier, unreliable data is highly deviant from the normal value, so we can classify user feedback into two types of service by assessing the user’s QoS values and deviation of normal values of reliable user clusters: Trusted feedback and untrusted feedback. Trusted feedback means that the QoS value of the service invoked by the user is within a normal range (trust interval), otherwise it is not trusted feedback.

The QoS value submitted by the user in the reliable interval would be closer to the normal value of service *j*. Thence, in order to obtain the trusted interval, similar to reference [40], we assume that the QoS values obtained from different users’ observations of the CPS service follow the Gaussian distribution N (μ, $\sigma^{2}$), where μ and σ represent the average of reliable user sets in each service and standard deviation, respectively. According to the 3σ rule [10] in the Gaussian normal distribution, there is a probability of 99.73% for users to observe QoS values in the range of [μ − 3σ, μ + 3σ], which can be set as the reliable interval of each service. Then user *i* invoking one of the services j in the trusted interval can be expressed as:

(3) $$nr_{i,j}=\{nr_{i,j}|\mu_{j}^{t}-3\sigma_{j}^{t}<nr_{i,j}\leq \mu_{j}^{t}+3\sigma_{j}^{t},nr_{i,j}\neq 0\}$$

where $\mu_{j}^{t}$ and $\sigma_{j}^{t}$ denote the average and standard deviation of users in the reliable interval *t* of service *j*, respectively, and $nr_{i,j}$ denotes the normalized QoS value after user *i* invokes service *j*.

According to the above steps, we calculate the credible interval of each service, then the user feedback information can be divided into trusted feedback, untrusted feedback, and no feedback, according to the QoS value of the CPS service invoked by the user. Finally, we count each user’s feedback information, and express the user’s feedback information as a feedback vector:

(4) $$\vec{Fb_{i}}=[po_{i},no_{i},ne_{i}]$$

where $\vec{Fb_{i}}$ denotes the feedback vector of user *i*, $po_{i}$ denotes the number of trusted feedback of user *i*, $no_{i}$ denotes the number of times without feedback, and $ne_{i}$ denotes the number of times of untrusted feedback.

#### 4.2.2. Calculating Users’ Reputations

User reputation reflects a measure of trustworthiness of the public about a user’s behavior feedback in the past. Reputation mechanisms can provide an incentive for honest feedback behavior and help users decide who is credible. In our GURAP approach, we calculate the user’s reputation (the QoS value that the user invokes the service in the future is the probability of the trusted feedback) by using the Dirichlet probability distribution, based on feedback from the user invoking the CPS service.

According to the user’s feedback, the probability of the trusted feedback of the user invoking the service in the future can be set as $p_{1}$, the probability of no feedback can be set as $p_{2}$, and the probability of untrusted feedback can be set as $p_{3}$. The probability that the user will invokes the service’s QoS feedback in the future is a vector $\vec{p}=(p_{1},p_{2},p_{3})$. Therefore, according to the Dirichlet probability distribution, the corresponding parameters are $\alpha_{1}$, $\alpha_{2}$, and $\alpha_{3}$, and the parameter vector can be represented as $\vec{\alpha }=(\alpha_{1},\alpha_{2},\alpha_{3})$. Therefore, the probability of the user’s future feedback can be expressed by the Dirichlet probability function $Dir(\vec{p}|\vec{\alpha })$ as follows:

(5) $$Dir(\vec{p}|\vec{\alpha })=\frac{\tau (\alpha_{1}+\alpha_{2}+...+\alpha_{K})}{\tau (\alpha_{1})...\tau (\alpha_{K})}\prod_{k}^{K}p_{k}^{\alpha_{k}-1},\begin{array}{cc} & K=3 \end{array}$$

where $p_{1}$ + $p_{2}$ + $p_{3}$ = 1, $\alpha_{1}$, $\alpha_{2}$, $\alpha_{3}$ > 0, and τ is a gamma function subjecting to $\tau (t)=\int_{0}^{\infty }x^{t-1}e^{-x}dx$. When the Dirichlet probability distribution parameter is an integer, τ(n)=(n−1)!. The probability expected value of the Dirichlet distribution can be given by the following:

(6) $$E(\vec{p})=(\frac{\alpha_{1}}{\sum_{i=1}^{K}\alpha_{i}},\frac{\alpha_{2}}{\sum_{i=1}^{K}\alpha_{i}},...,\frac{\alpha_{K}}{\sum_{i=1}^{K}\alpha_{i}}),\begin{array}{cc} & K=3 \end{array}$$

The user’s feedback before the user invokes CPS service is unknown, but the sum of probabilities for all cases equals one. Therefore, the prior probability of the user’s various feedback conditions should obey uniform distribution Uniform (0,1). Therefore, a variety of feedback of users invoking CPS services conforms to the Dirichlet distribution $Dir(\vec{p}|\vec{1})$. As described above, after the user invokes the service, the number of positive feedback after the user *i* invokes the CPS service is $po_{i}$, the number of no feedback is $no_{i}$, the number of negative feedback is $ne_{i}$, and the count vector can be set to $\vec{m}=(po_{i},no_{i},ne_{i})$. The posterior distribution of the feedback probability $\vec{p}$ of user *i* can be expressed as follows, because the counting vectors conform to a multinomial distribution:

(7) $$Dir(\vec{p}|\vec{1}+\vec{m})=\frac{\tau (po_{i}+no_{i}+ne_{i}+3)}{\tau (po_{i}+1)\tau (no_{i}+1)\tau (ne_{i}+1)}\cdot (p_{i,1}^{{\mathrm{po}}_{i}}p_{i,2}^{no_{i}}p_{i,3}^{ne_{i}})$$

According to the Dirichlet probability distribution property, when the feedback probability $\vec{p}$ of the service invoked by the user is the probability expectation of Dirichlet distribution, the Dirichlet probability distribution has the highest relative frequency. That is to say, the value of probability $\vec{p}$ of the user is most likely to be the probability expectation of the Dirichlet distribution. Therefore, once the user’s various feedback is known, the probability of trusted feedback, non-feedback, and untrusted feedback of the future invocation service of the user *i* can be expressed as follows:

(8) $$\begin{array}{l} p_{i,1}=E(Dir(\vec{p}|\vec{1}+{\vec{m}}_{i}))=\frac{po_{i}+1}{po_{i}+no_{i}+ne_{i}+3}\\ p_{i,2}=E(Dir(\vec{p}|\vec{1}+{\vec{m}}_{i}))=\frac{no_{i}+1}{po_{i}+no_{i}+ne_{i}+3}\\ p_{i,3}=E(Dir(\vec{p}|\vec{1}+{\vec{m}}_{i}))=\frac{ne_{i}+1}{po_{i}+no_{i}+ne_{i}+3} \end{array}$$

where $p_{i,1}$, $p_{i,2}$, and $p_{i,3}$ denote the probabilities of trusted feedback, no feedback, and untrusted feedback of the user *i* invoking services in the future, respectively. This is because we can only evaluate whether the user is credible when the user invokes the service and has feedback, namely the user’s reputation. That is to say, the user’s reputation is the probability of trusted feedback when the user has feedback. Therefore, the user’s reputation can be expressed as:

(9) Rei=pi,1pi,1+pi,3,(pi,1+pi,3)≠0

where Rei denotes the reputation of user *i* and its value is between [0, 1], because the probability of user’s trusted feedback is between [0, 1]. For example, after the user *i* invokes the CPS service, the number of trusted feedback is 7, the number of non-feedback is 22, and untrusted feedback number is 1. Therefore, the probability of trusted feedback of the user invoking the service in the future is $p_{i,1}$ = $\frac{7+1}{7+22+1+3}\approx 0.24242$, the probability of no feedback is $p_{i,2}$ = $\frac{22+1}{7+22+1+3}\approx 0.69697$, and the probability of untrusted feedback is $p_{i,3}$ = $\frac{1+1}{7+22+1+3}\approx 0.06061$. Finally, the reputation value of user *i* is Rei = $\frac{p_{i,1}}{p_{i,1}+p_{i,3}}=\frac{0.24242}{0.06061+0.24242}\approx 0.79999$.

#### 4.2.3. Identifying Untrustworthy Users and Fixing Unreliable QoS Values

(1) Identifying untrusted users

Based on the Dirichlet probability distribution and the QoS value of the user invoking services, we can calculate the reputation value of all users, then we can identify the untrusted users by the user’s reputation value. In our approach, the approach of user reputation-aware CURA is to identify untrustworthy users by using the thresholds δ. When the user’s reputation value is less than threshold δ, the user is identified as untrustworthy. Identifying untrusted users is given by the following formula:

(10) U¯={u|Reu<δ}

where $\bar{U}$ is the set of untrusted users and δ denotes the threshold that can be used to identify untrusted users. Our approach is to calculate the appropriate threshold δ in the experiment, which can be directly used to identify untrusted users. However, in the real world, the threshold δ is still very difficult to set, because when the threshold δ is set to be low, untrusted users may be identified as credible, and this could lead to a lower prediction accuracy. When the threshold δ is set to be very high, it may also identify trusted users as untrustworthy, and affect the predictive performance of our approach. A comprehensive experimental study of thresholds will be conducted in Section 5.

(2) Fixing unreliable QoS values

In the real world, the collected QoS data is very sparse. If QoS values provided by untrusted users are directly filtered, the QoS matrix will be sparser. Hence, our approach is to identify the untrusted users by user reputation and then fix the untrusted data provided by untrusted users. The formula for unreliable data correction can be expressed as follows:

(11) $$r_{u,j}^{\prime }=\{\begin{array}{ccc} \bar{Ru_{j}^{\max}} & , & r_{u,j}>0\\ 0 & , & r_{u,j}=0 \end{array}\;\mathrm{and}\;u\in \bar{U}$$

where $r_{u,j}^{\prime }$ denotes the modified QoS value of untrusted user u on service *j*, $r_{u,j}$ denotes the original QoS value of the untrusted user *u* in service *j*, and $\bar{Ru_{j}^{\max}}$ denotes the mean of the QoS values (this is not the normalized QoS value of the matrix R) of the user set $U_{j}^{\max}$ in the reliable interval of service *j*. Modifying the unreliable QoS data contributed by untrusted users can get a new m×n QoS matrix $R^{\prime }$, where $r_{i,j}^{\prime }$ is the QoS value of user *i* invoking service *j*.

### 4.3. Geographic-Based User (Service) Similarity Neighbor Recognition

Experiments based on reference [18] show that the rating of services has different positive correlation degrees with the average rating of its neighbors, for different geographic regions levels, and the vast majority of services have neighbors under the geographic region-level relationship. It is shown in reference [9] that users in the same geographical area tend to have similar performance, so it is highly valuable to take the information of user location into account for QoS prediction. Therefore, this paper digs up potential geographical position information of user services to better identify similar geographical neighbors of user services and alleviate the data matrix sparseness. Our approach, GUIPCC, identifies the user services similarity geographical neighbor according to the following three steps: The geographic neighborhood stratification, computation of geographic neighborhood correlation-degree, and the identification of similar geographical neighbors of user services.

#### 4.3.1. The Geographic Neighborhood Stratification

In this paper, we consider the geographical information of each user from three aspects: The latitude and longitude, country and autonomous network (AS), and the geographical information of each service from the latitude and longitude, AS, and provider. We think geographical coordinates are not enough for two reasons: First, the literature [41] indicates that simply because users have close coordinates does not mean that they are definitely in the same AS network, while the network environments are similar within an AS network, and different among multiple AS networks. Therefore, it is valuable to divide users/services according to their AS networks, so as to capture the users/services’ similarity within an AS network. Similarly, we also capture the similarity within a country. Second, when we only consider geographical coordinates, the prediction performance of clustering users according to coordinates is not satisfying, since there are only a few users in each group, so we employ the country level and AS level of similar users to solve the inaccuracy caused by data sparseness.

For the stratification of geographical neighborhood of services, we employ a two-point K-means clustering algorithm to cluster the latitude and longitude of the service to obtain $K_{S}$ service clusters. For service $S_{j}$, we divide the services into $K_{S}$ service clusters by minimizing the following formula:

(12) $$SJ=\sum_{k=1}^{K}\sum_{{\vec{L}}_{j}\in C_{k}}{\left\|{\vec{L}}_{j}-{\vec{\mu }}_{k}\right\|}^{2},\begin{array}{cc} \mathrm{and} & \begin{array}{cc} 1\leq K\leq K_{S} & \end{array} \end{array}$$

where $C_{k}$ denotes the kth service cluster in the service, ${\vec{L}}_{j}$ denotes the latitude and longitude vector ${\vec{L}}_{j}=(Lo_{j},La_{j})$ of the service $S_{j}$, ${\vec{\mu }}_{k}$ denotes the center point of the *k*th service cluster, and SJ denotes the sum of squared errors of the kth iteration of the two-point K-means algorithm. The two-point K-means clustering algorithm makes the SJ minimum at each iteration, and it does not stop until the number of service clusters reaches the expected $K_{S}$ service cluster. Therefore, the two-point K-means does not only exist on a random centroid selection, but also minimizes the sum of square error of each step.

**Figure 3:**
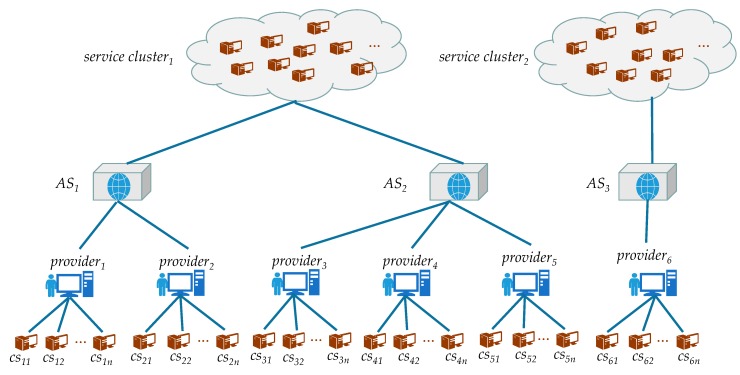
Hierarchical relationship of *Provider-Level*, *SAS-Level*, and *SCluster-Level*.

Because the users’ number in the dataset is too small, we set all users as one user cluster. By clustering, we get user clusters and service clusters, respectively. We classify the users in the users cluster into different country geographic neighborhood levels, according to the user’s country attribute, and then classify the users in the country geographic neighborhood through the user’s AS attributes into different AS geographic neighborhood levels. Similarly to users, we classify the services in a service cluster into multiple AS geographic neighborhoods and classify the services in the AS geographic neighborhood into multiple provider geographic neighborhoods. In this way, we divide the users and services into three-tier geographic neighborhoods levels, respectively. For example, the relationship between the three-tier geographic neighborhoods level is shown in Figure 3.

We define *Provider-Level*, *SAS-Level*, and *SCluster-Level* as the CPS service groups that are geographically close to the target CPS service at the corresponding zone level, and then define *UAS-Level*, *Country-Level*, and *UserSet-Level* as geographic user groups near the target user. The user’s *UserSet-Level* represents a collection of all users. Service providers *Provider-Level*, service autonomous network *SAS-Level*, user autonomous network *UAS-Level*, and user country *Country-Level* denote that services or users with the same geographic characteristics are clustered in the same area level. For example, services with the same CPS service provider are grouped together in the same *Provider-Level* area.

We set the geographical neighbor cluster of the *UAS-Level* area where the target user a is located as $AN_{a}^{u}$, the geographical neighbor cluster in the *Country-Level* area where the target user a is located but not in *UAS-Level* area as $CoN_{a}^{u}$, the geographical neighbor user cluster of the target user a in the *Country-Level* area as $ACoN_{a}^{u}$, and the geographical neighbor user cluster that is not in the *Country-Level* area where the target user a is located as $ClN_{a}^{u}$.

We set the geographical neighbor service cluster of the *Provider-Level* region where the target service t is located as $PN_{t}^{s}$, the geographical neighbor service cluster in the *SAS-Level* region where the target service t is located but not in the *Provider-Level* region as $AN_{t}^{s}$, the geographical neighbor service cluster of the target service t in the *SAS-Level* region as $APN_{t}^{s}$, and the geographical neighbor service cluster in the *SCluster-Level* area where the target service t is located but not in the *SAS-Level* area as $CN_{t}^{s}$.

#### 4.3.2. Calculating Geographic Neighborhood Correlation Degree

To dig up the relationships among users/services in locations so as to identify their geographical neighbors, the geographic neighborhood correlation-degree of users/services needs to be computed. For three-tiered geographic neighbors of users/services, we only need to compute the correlation among users/services in a user/service cluster. We first give the PCC similarity calculation formula for users/services:

(13) $$sim(a,u)=\{\begin{array}{cc} \frac{\sum_{j\in S}(r_{a,j}^{\prime }-\bar{r_{a}^{\prime }})(r_{u,j}^{\prime }-\bar{r_{u}^{\prime }})}{\sqrt{\sum_{j\in S}(r_{a,j}^{\prime }-\bar{r_{a}^{\prime }}{)}^{2}}\sqrt{\sum_{j\in S}{\left(r_{u,j}^{\prime }-\bar{r_{u}^{\prime }}\right)}^{2}}}, & \left|S\right|>1\\ 0, & 0\leq \left|S\right|\leq 1 \end{array}$$

(14) $$sim(t,s)=\{\begin{array}{cc} \frac{\sum_{i\in U}\left(r_{i,t}^{\prime }-\bar{r_{t}^{\prime }})(r_{i,s}^{\prime }-\bar{r_{s}^{\prime }}\right)}{\sqrt{\sum_{i\in U}{\left(r_{i,t}^{\prime }-\bar{r_{t}^{\prime }}\right)}^{2}}\sqrt{{\sum_{i\in U}\left(r_{i,s}^{\prime }-\bar{r_{s}^{\prime }}\right)}^{2}}}, & \left|U\right|>1\\ 0, & 0\leq \left|U\right|\leq 1 \end{array}$$

where sim(a,u) denotes the similarity between user a and u, S denotes the CPS service set previously invoked by user a and u, |S| denotes the number of services invoked by user a, and u, $\bar{r_{a}^{\prime }}$, and $\bar{r_{u}^{\prime }}$ denote the average QoS value of user a and u invoking the CPS service in QoS matrix $R^{\prime }$, respectively. sim(t,s) denotes the similarity between services t and s. U denotes the set of users that have previously invoked CPS services t and s, |U| denotes the number of users invoking CPS services t and s, $r_{i,t}^{\prime }$ denotes the QoS value of user i invoking service t in the user service QoS matrix $R^{\prime }$, and $\bar{r_{t}^{\prime }}$ and $\bar{r_{s}^{\prime }}$ represent the average QoS value of the user accessing the services t and s, respectively.

According to Formulas (13) and (14), we first calculate the similarity between users in the user cluster, and services in the service cluster. Then we calculate the average similarity $\bar{AU_{a}}$ between user a and users in $AN_{a}^{u}$, average similarity $\bar{CoU_{a}}$ between user a and users in $CoN_{a}^{u}$, average similarity $\bar{ClU_{a}}$ between user a and users in $ClN_{a}^{u}$, and average similarity $\bar{ACoU_{a}}$ between user a and users in $ACoN_{a}^{u}$. Then we calculate the average similarity $\bar{PS_{t}}$ between service t and services in $PN_{t}^{s}$, average similarity $\bar{AS_{t}}$ between service t and services in $AN_{t}^{s}$, average similarity $\bar{APS_{t}}$ between service t and services in $APN_{t}^{s}$, and average similarity $\bar{CS_{t}}$ between service t and services in $CN_{t}^{s}$.

The positive correlation is different due to the service at different geographical neighborhood levels, and in order to better assess similar users and service geographical neighbors, we evaluate the correlation between the three-tier geographical neighborhood levels of users or services. We set the geographic correlation-degree $\gamma_{a}^{co}$, $\gamma_{a}^{cl}$ of the two users, and set the geographic correlation-degree $\varphi_{t}^{a}$, $\varphi_{t}^{c}$ of the two services. The correlation-degree formula can be expressed as:

(15) $$\gamma_{a}^{co}=\{\begin{array}{cc} \frac{\bar{CoU_{a}}}{\bar{AU_{a}}}, & \bar{CoU_{a}}>0\wedge \bar{AU_{a}}>0\wedge \bar{AU_{a}}>\bar{CoU_{a}}\\ 1, & otherwise \end{array}$$

(16) $$\gamma_{a}^{cl}=\{\begin{array}{cc} \frac{\bar{ClU_{a}}}{\bar{ACoU_{a}}}, & \bar{ClU_{a}}>0\wedge \bar{ACoU_{a}}>0\wedge \bar{ACoU_{a}}>\bar{ClU_{a}}\\ \frac{\bar{ClU_{a}}}{\bar{AU_{a}}}, & \bar{ClU_{a}}>0\wedge \bar{AU_{a}}>0\wedge \bar{AU_{a}}>\bar{ClU_{a}}\\ 1, & otherwise \end{array}$$

(17) $$\varphi_{t}^{a}=\{\begin{array}{cc} \frac{\bar{AS_{t}}}{\bar{PS_{t}}}, & \bar{AS_{t}}<\bar{PS_{t}}\wedge \bar{PS_{t}}>0\wedge \bar{AS_{t}}>0\\ 1, & otherwise \end{array}$$

(18) $$\varphi_{t}^{c}=\{\begin{array}{cc} \frac{\bar{CS_{t}}}{\bar{APS_{t}}}, & \bar{APS_{t}}>0\wedge \bar{CS_{t}}>0\wedge \bar{APS_{t}}>\bar{CS_{t}}\\ \frac{\bar{CS_{t}}}{\bar{PS_{t}}}, & \bar{PS_{t}}>0\wedge \bar{CS_{t}}>0\wedge \bar{PS_{t}}>\bar{CS_{t}}\\ 1, & otherwise \end{array}$$

where $\gamma_{a}^{co}$ denotes the correlation-degree between the user cluster $CoN_{a}^{u}$ and the user cluster $AN_{a}^{u}$, and $\gamma_{a}^{cl}$ denotes the correlation-degree between the service cluster $\bar{ClU_{a}}$ and the service cluster $ACoN_{a}^{u}$. $\varphi_{t}^{a}$ denotes the correlation-degree between the service cluster $AN_{t}^{s}$ and the service cluster $PN_{t}^{s}$, and $\varphi_{t}^{c}$ denotes the correlation-degree between the service cluster $CN_{t}^{s}$ and the service cluster $APN_{t}^{s}$.

#### 4.3.3. The Identification of Similar Geographical Neighbors of Users and Services

In the real world, when the user service QoS matrix is sufficiently sparse, the target user and the user in the *UserSet-Level* geographical area may not have a service to be observed jointly, which results in the target user having little or no similar geographical neighbors, thereby reducing the QoS prediction accuracy. In order to ensure that the user has enough similar geographical neighbors, the similarity that the user doesn’t commonly invoke a service with target users is replaced with the average similarity of the target user’s geographical neighborhood level. The modified similarity formula is as follows:

(19) $$sim_{u}(a,u)=\{\begin{array}{c} \bar{AU_{a}},\begin{array}{ccc} & & u\in AN_{a}^{u}\wedge sim(a,u)=0 \end{array}\\ \bar{CoU_{a}},\begin{array}{ccc} & & u\in CoN_{a}^{u}\wedge sim(a,u)=0 \end{array}\\ \bar{ClU_{a}},\begin{array}{ccc} & & u\in ClN_{a}^{u}\wedge sim(a,u)=0 \end{array} \end{array}$$

Experiments in [18] show that the services have different neighbor qualities at different regional levels, and there is a stronger positive correlation between the services in *Provider-Level* geographic regions than those in *SAS-Level* and *SCluster-Level* regions. The same positive correlation between users in different user neighborhood levels is also different. Therefore, in order to make the similar neighbors of more users and services in a stronger positive correlation geographical neighborhood level, we improve the user and service similarity by the correlation-degree between geographical neighborhood levels. The improved service similarity formula can be expressed as:

(20) $$sim_{u}^{\prime }(a,u)=\{\begin{array}{ll} sim_{u}(a,u), & u\in AN_{a}^{u}\\ sim_{u}(a,u)\times \gamma_{a}^{co}, & u\in CoN_{a}^{u}\\ sim_{u}(a,u)\times \gamma_{a}^{cl}, & u\in ClN_{a}^{u} \end{array}$$

(21) $$sim_{s}^{\prime }(t,s)=\{\begin{array}{ll} sim(t,s), & s\in PN_{t}^{s}\\ sim(t,s)\times \varphi_{t}^{a}, & s\in AN_{t}^{s}\\ sim(t,s)\times \varphi_{t}^{c}, & s\in CN_{t}^{s} \end{array}$$

According to the above steps, we calculate the final similarity between the target user and all users in *UserSet-Level*, and the similarity between the target service and all the services of *SCluster-Level* where the target service resides. Next, we use the traditional Top-k algorithm to identify the top $K_{NU}$ similar geographic neighbors of the target user and the top $K_{NS}$ similar geographic neighbors of the target service, respectively. Then similar neighbors, whose similarities are equal to or less than 0, are filtered out. Finally, we use N(u) to represent the set of similar geographical neighbors of the target user, while N(s) represents the set of similar geo-neighbors for the target service.

### 4.4. Unknown QoS Value Prediction Based on Reliable Data

We identified untrusted users in Section 4.2 and obtained similar geographic neighbors for services and users in Section 4.3. Hence, we can calculate the unknown QoS value of the trusted users, based on the similar geographic neighbors of the trusted user and the new QoS matrix. The formula for the unknown QoS prediction is as follows:

(22) $${\hat{r}}_{u,s}=\bar{r_{u}^{\prime }}+\frac{\sum_{u^{\prime }\in N(u)}{sim}_{u}^{\prime }(u,u^{\prime })(r_{u^{\prime },s}^{\prime }-\bar{r_{u^{\prime }}^{\prime }})}{\sum_{u^{\prime }\in N(u)}{sim}_{u}^{\prime }(u,u^{\prime })}$$

where u denotes the user identified as trusted, ${\hat{r}}_{u,s}$ represents the predicted value of user u on service s, $\bar{r_{u}^{\prime }}$ represents the average QoS value observed by user u for all services in the $R^{\prime }$ matrix, N(u) denotes the user set of similar geographical neighbors of user u, and ${sim}_{u}^{\prime }(u,u^{\prime })$ represents the similarity between user u and similar user $u^{\prime }$.

Since the user service QoS matrix is very sparse in the real world, it is likely that the QoS value $r_{u^{\prime },s}^{\prime }$ that the similar neighbor $u^{\prime }$ of user u observes the service s to be equal to 0. If the QoS value that the majority similar neighbor $u^{\prime }$ of user u invokes service s equals to 0, the accuracy of QoS value prediction will be reduced. Therefore, in order to mitigate the effect of the sparseness of the QoS matrix, an approximation of the service s to be invoked by the user $u^{\prime }$ can be substituted when $r_{u^{\prime },s}^{\prime }$ equals to 0. $r_{u^{\prime },s}^{\prime }$ is calculated as follows:

(23) $$r_{u^{\prime },s}^{\prime }=\{\begin{array}{ll} \bar{r_{s}^{\prime }}+\frac{\sum_{s^{\prime }\in N(s)}{sim}_{s}^{\prime }(s,s^{\prime })(r_{u^{\prime },s^{\prime }}^{\prime }-\bar{r_{s^{\prime }}^{\prime }})}{\sum_{s^{\prime }\in N(s)}{sim}_{s}^{\prime }(s,s^{\prime })}, & r_{u^{\prime },s}^{\prime }=0\\ r_{u^{\prime },s}^{\prime } & r_{u^{\prime },s}^{\prime }\neq 0 \end{array}$$

where $\bar{r_{s}^{\prime }}$ and $\bar{r_{s^{\prime }}^{\prime }}$ denote the average QoS values of services *s* and *s*′ observed by the users in the QoS matrix *R*′, respectively, $r_{u^{\prime }s^{\prime }}^{\prime }$ denotes the QoS value of the service *s*′ invoked by the user *u*′ in the QoS matrix *R*′, *N*(*s*) denotes the service set of similar geography neighbors of the service *s*, and ${sim}_{s}^{\prime }(s,s^{\prime })$ represents the similarity of service *s* with its similar geographical neighbor *s*′. By using Formulas (22) and (23), we can predict the unknown QoS value of trusted users on the CPS service.

## 5. Experiments Results

In this section, we conduct a broad experimental evaluation of the proposed GURAP approach. The experimental and other approaches we proposed are implemented with the jupyter notebook editor in Python, and run on a machine with an Intel ^®^ Core ™ i5-4590 CPU @ 3.30GHZ processor, 8GB of RAM, and Windows 7 Sp1 system. The experiment includes two parts: (1) Comparison of GURAP with other popular approaches; and (2) verification of the influence of different parameters on prediction accuracy.

### 5.1. Experimental Settings

The dataset in experiments comes from the real dataset published by Zheng et al. [42,43,44], including two matrices, the response time matrix (rtMatrix), and the throughput matrix(tpMatrix), both of which contain real QoS assessment results from 339 users of 5825 services. The dataset collects 1,974,675 response-time and throughput records. Response times range from 0 to 20 s, and the throughput ranges from 0 to 1000 kbps. This data set also contains the geographical location information of the user side and service side. Geographic location information includes geographical information and network location information. Geographic information includes the latitude and longitude of users and services, and the country to which the user belongs. Network location information includes the AS (Autonomous Network) and service providers that include the service. In our experiments, we use the response time dataset and randomly set p% of the users as untrusted users, and QoS values contributed by untrusted users will be replaced by randomly generated values. To meet the requirement of matrix sparsity in the real world, we randomly remove the entries in the QoS matrix to a certain density and then use the removed QoS entry as the expected value to study the prediction performance.

### 5.2. Experimental Indicators

The first experimental evaluation indicator is the identification of untrusted users accuracy (IUUA), which assesses the accuracy of our approach in identifying untrusted users and proves the validity of our approach. It is defined as follows:

(24) $$IUUA=\frac{2\times \left|U_{I}^{T}\right|}{\left|U_{I}^{A}\right|+\left|U_{T}\right|}$$

where $\left|U_{I}^{T}\right|$ indicates the number of genuine untrustworthy users after our approach identification, $\left|U_{I}^{A}\right|$ means the number of untrusted users identified through our approach, and $\left|U_{T}\right|$ represents the number of untrusted users we randomly set. The larger the IUUA value is, the better the recognition accuracy is. When the value is equal to 1, it indicates that all the untrusted users in the QoS data are identified, and none of the trusted users are identified as untrustworthy.

The mean absolute error (MAE) and root mean squared error (RMSE) metrics are evaluation indicators commonly used to evaluate the prediction accuracy of QoS prediction methods in CF. Smaller MAE and RMSE values indicate a higher accuracy, which is defined as follows:

(25) $$MAE=\frac{\sum_{u,s}\left|r_{u,s}-{\hat{r}}_{u,s}\right|}{N}$$

(26) $$RMSE=\sqrt{\frac{\sum_{u,s}{\left|r_{u,s}-{\hat{r}}_{u,s}\right|}^{2}}{N}}$$

where N is the number of missing entries that need to be predicted in the QoS matrix, $r_{u,s}$ is the QoS value of the trusted user u observing the service s, and ${\hat{r}}_{u,s}$ represents the predicted QoS value. Since QoS value ranges for different services are different, we use a normalized mean absolute error (NMAE) metric to measure the prediction accuracy, where a smaller NMAE value implies higher prediction accuracy. NMAE is defined as:

(27) $$NMAE=\frac{MAE}{\sum_{u,s}r_{u,s}/N}$$

### 5.3. Comparison

In this section, to demonstrate the validity of our GURAP approach based on geographic location and reputation-aware, we conducted extensive experiments with the most advanced QoS prediction approaches and compared our approach to them. We select several of the following approaches to compare with our approach.

- UPCC: In this approach, the QoS value is predicted by utilizing the similarity between two users using the Pearson correlation coefficient (PCC), and the history invocation records of similar users [45].
- IPCC: This approach is similar to the UPCC method, except that it predicts the unknown QoS value by calculating the similarity between two services and using the QoS value of similar services [46].
- UIPCC: This approach integrates the UPCC and IPCC approaches into a unified model, and then uses the similar users and similar services to combine their predicted results with specific parameters *λ* [33].
- CURA: This approach is the one we proposed in Section 4.2 of this paper that predicts unknown QoS entries by identifying untrusted users and processing unreliable data and then combining the processed QoS data with the UPCC method.
- GUIPCC: GUIPCC identifies similar services and similar users using the QoS data of unidentified untrusted users by the approach proposed in Section 4.2 of this article, and then uses the prediction approach in UIPCC to predict missing QoS values.
- TAP: In this approach, user reputation is evaluated and a set of reliable similar users are identified by using K-means clustering and Beta distribution, and then the results predicted by similar users and the unknown QoS values predicted by similar services, based on service clustering, are combined with specific parameters *λ* [17].
- GNMF: In this approach, similar service geo-neighbors are identified by using a bottom-up hierarchical neighborhood clustering of service geography and then integrated into matrix decomposition to predict [18].

In our experiments, matrix density is defined as the density of the original QoS data set. Since the matrices in the real world are usually very sparse, we investigate the effect of different matrix densities on the prediction accuracy of our method, where the step size is 5%, changing the QoS matrix density from 5% to 30%. The percentage of untrusted users is set to 2.95% and 5.90%, indicating that the randomly selected 2.95% or 5.90% of the 339 users are set as untrusted users. *UK* is set to 15 and the statistical interval in our GURAP approach is set to 15. *δ* is set to 0.10, indicating that the threshold for identifying untrusted users is 0.10. *K_S_* is set to 9, indicating that the service is clustered into nine service clusters through latitude and longitude clustering. *K_NS_* and *K_NU_* are set to 35 and 12, respectively, indicating that the 35 most similar services and the 12 most similar users geo-neighbors are selected for prediction

Table 2 and Table 3 show the NMAE and RMSE results for different approaches at different densities for two percentages of untrusted users, while the bold ones indicate the result of our approach. The experimental results show several important results.

(1) In different response time matrix densities, our approach can achieve smaller NMAE and RMSE values than other approaches at different response time matrix densities, which shows that our approach is more accurate than the existing approaches, in most cases. Specifically, compared with TAP, in the case of 2.95% untrusted users, the values of NMEAE and RMSE in our approach GURAP increased by an average of 16.83% and 17.05%, respectively. In the case of 5.90% of untrustworthy users, they increased by an average of 17.49% and 17.83%, respectively. Compared with the GNMF model in the case of two kinds of untrustworthy users, in GURAP the NMAE values reached 14.62% and 13.32%, respectively, while the RMSE values reached 26.19% and 27.59%, respectively.
(2) Compared with UPCC, IPCC, UIPCC, and GUIPCC, our approach GURAP achieves better prediction accuracy. The reason for this result is that our approach guarantees the reliability of the data by identifying untrusted users through the user reputation method and by using the average QoS value of the reliable users to correct the value contributed by the untrusted user. Other approaches do not consider whether the QoS data provided by the user is reliable. As the GUIPCC approach considers the geographical location information, the prediction accuracy is improved, compared to the other three approaches.
(3) Compared with the CURA and TAP approaches, our approach mines the potential geographical information relationships of users and services so that more similar neighbors can be obtained and geographical neighbors of users can be used to mitigate the matrix sparseness. TAP does not consider the impact of geographical location, but also ignores that the contribution of untrusted users will affect the prediction accuracy, therefore as the matrix density increases, the prediction accuracy decreases. Because the CURA method processes the unreliable data, as the matrix density increases, the prediction accuracy can continue to improve.
(4) Compared with the GNMF method, our approach achieves smaller NMAE and RMSE values in QoS prediction, but the degree of reduction in RMSE value is very large, because the RMSE evaluation index is more sensitive to outliers. Because our approach identifies untrustworthy users and processes the values of unreliable data, while GNMF does not consider unreliable data and directly uses the original QoS data to predict., our approach has better prediction accuracy than GNMF.
(5) When the response time matrix density is 5%, the GURAP approach’s NMAE value is similar to the TAP method, and the RMSE value in the TAP method is greater than the GURAP approach, because in the TAP method, when the data is very sparse, the volume of untrusted data is also very small, therefore the impact of untrusted users on QoS prediction is small. However, the RMSE assessment index is very sensitive to outliers, and the TAP method does not processes untrusted data, so the RMSE value is larger than in GURAP. When the matrix density is greater than 5%, our approach is greatly improved compared to the TAP method. Compared with GNMF, The prediction accuracy of the GURAP approach is always larger than that of GNMF, and increases as matrix density increases. Experiments show that our approach can achieve better prediction accuracy by considering user reputation and the potential geographic information of service users.

### 5.4. Impacts of Percentage of Untrusted Users

**Figure 4:**
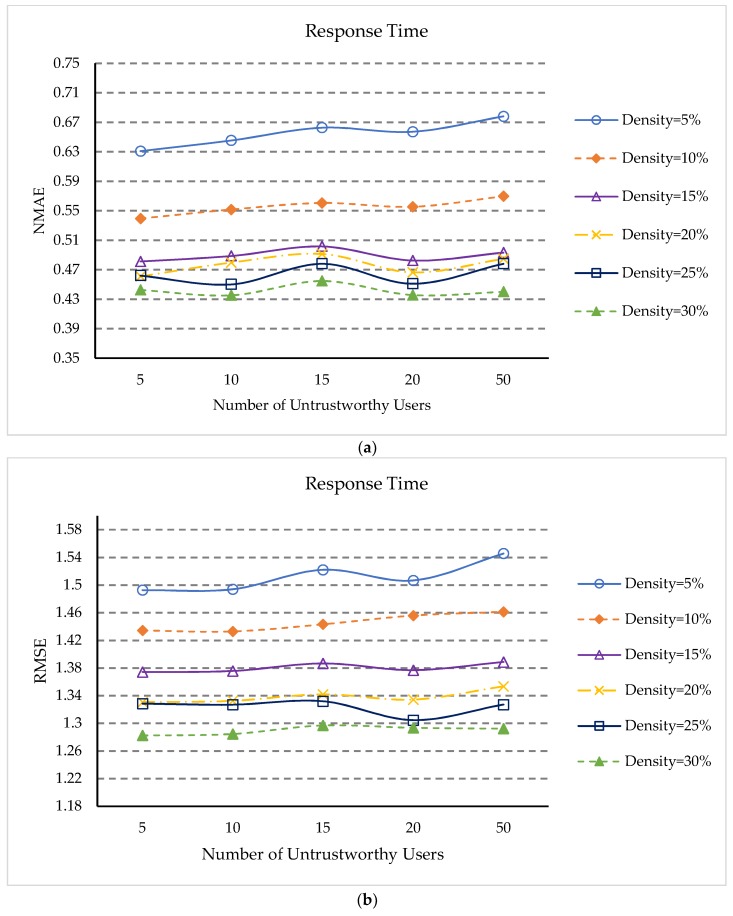
(**a**) Impact of untrustworthy users (NMAE); (**b**) Impact of untrustworthy users (RMSE).

In order to study the impact of the percentage of untrusted users on prediction accuracy, we set the number of untrusted users to 5, 10, 15, 20, and 50, and the percentage of untrusted users to 1.47%, 2.95%, 4.42%, 5.90%, and 14.75%, respectively. The matrix densities vary from 5% to 30%, and *δ*, *UK*, *K_S_*, *K_NS_*, and *K_NU_* parameter settings are the same as Section 5.3. The experimental results are shown in Figure 4.

Experimental results show:

(1) In Figure 4a,b, when the percentage of untrustworthy users is 1.47%, the QoS prediction assessment indicators NMAE and RMSE are generally the smallest. When the matrix density is small, the overall trend is slowly increasing, but their NMAE and RMSE values are all very close. This is because the data obtained by our approach when processing untrusted data is only close to the true value, when the number of untrusted users increases, the increase of untrusted data will affect the prediction accuracy of QoS. However, when the matrix density is relatively large, the NMAE and RMSE values fluctuate within a certain range. This indicates that our method can identify unreliable users and can handle untrusted QoS data.
(2) From Figure 4a,b, it can also be seen that NMAE and RMSE continue to reduce as the matrix density becomes more and more dense. This is because for different percentages of untrusted users, more dense matrices can provide more information.

### 5.5. Impact of Threshold δ

**Figure 5:**
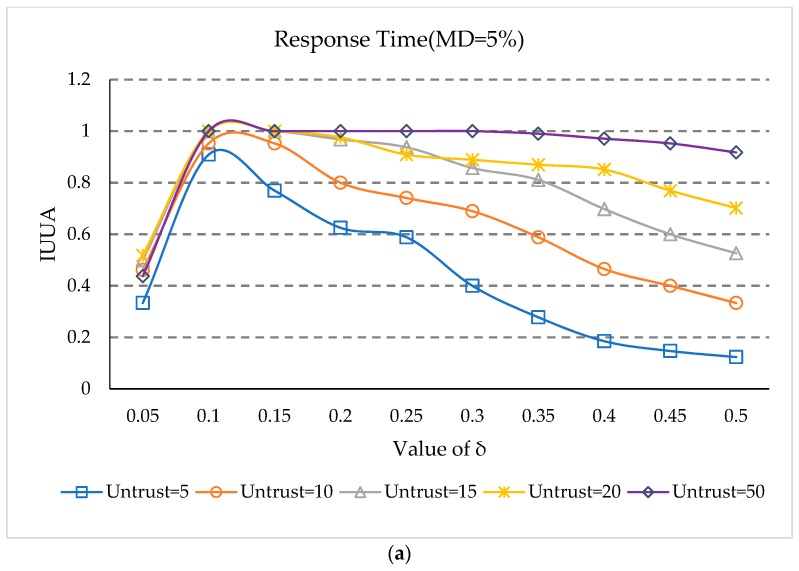

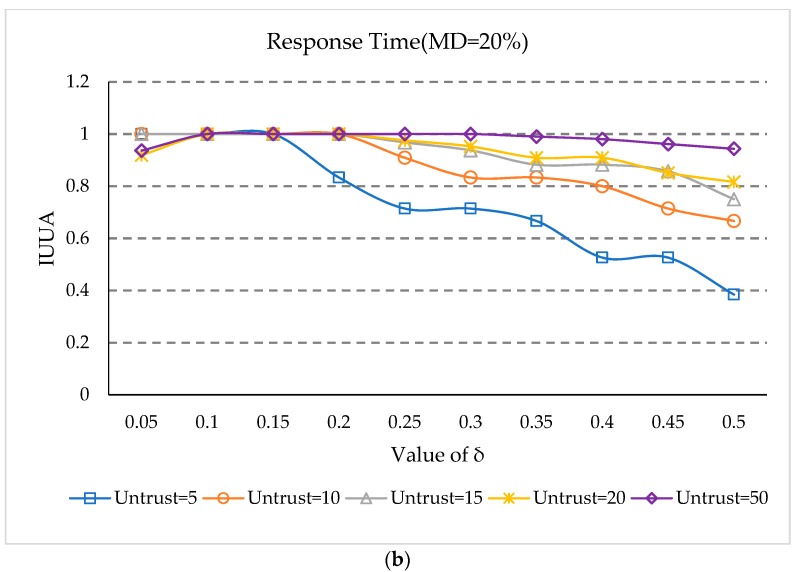
(**a**) Impact of Threshold *δ* (MD = 5%); (**b**) Impact of Threshold *δ* (MD = 20%).

*δ* is a threshold for identifying an untrusted user, and when the user reputation value is less than this threshold, the user is identified as an untrusted user. To study the effect of thresholds on identifying the untrusted users, we varied the threshold *δ* from 0.05 to 0.50 in steps of 0.05. The number of untrusted users is set to 5, 10, 15, 20, and 50 and the statistical interval *UK* is 15. This experiment is conducted at 5% and 20% matrix densities. The experimental results are shown in Figure 5.

Figure 5 shows the impact of thresholds on identifying untrusted users. We make a few observations.

(1) At first, IUUA increases rapidly as *δ* increases, when the matrix density is 5%. This is because user reputation methods cannot identify untrusted users when *δ* is small. After IUUA rises to 1, it declines as the value of *δ* increases, because when the value is too large, trusted users will be identified as untrusted users.
(2) In Figure 5a,b, there are the earliest declines in the case of five untrusted users, and the slowest drop for 50 untrusted users. This is because when the number of untrusted users is small, some feedback situations of the trusted users can be easily recognized as untrusted feedback, as some trusted users have relatively small reputation values. Therefore, with *δ* increasing, trusted users can easily be identified as untrusted users, which influences the prediction accuracy.
(3) It can be seen from Figure 5 that when the value is 0.10, the IUUA value can be maximized at both matrix densities.

### 5.6. Impact of UK

**Figure 6:**
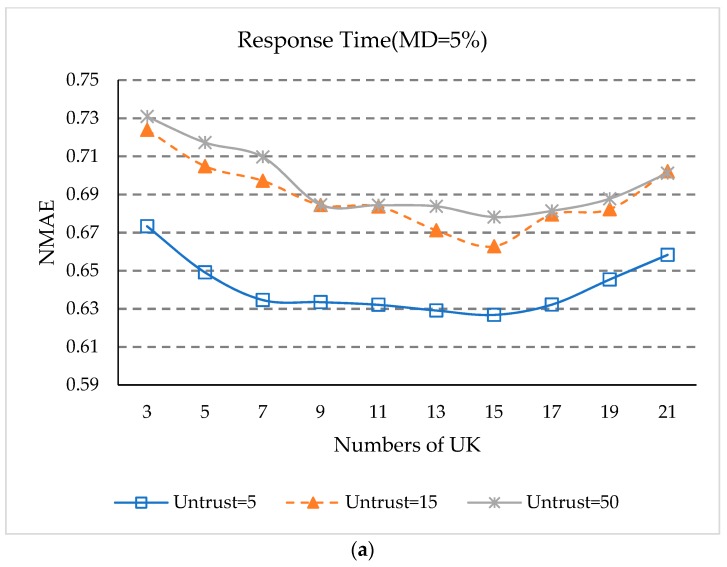

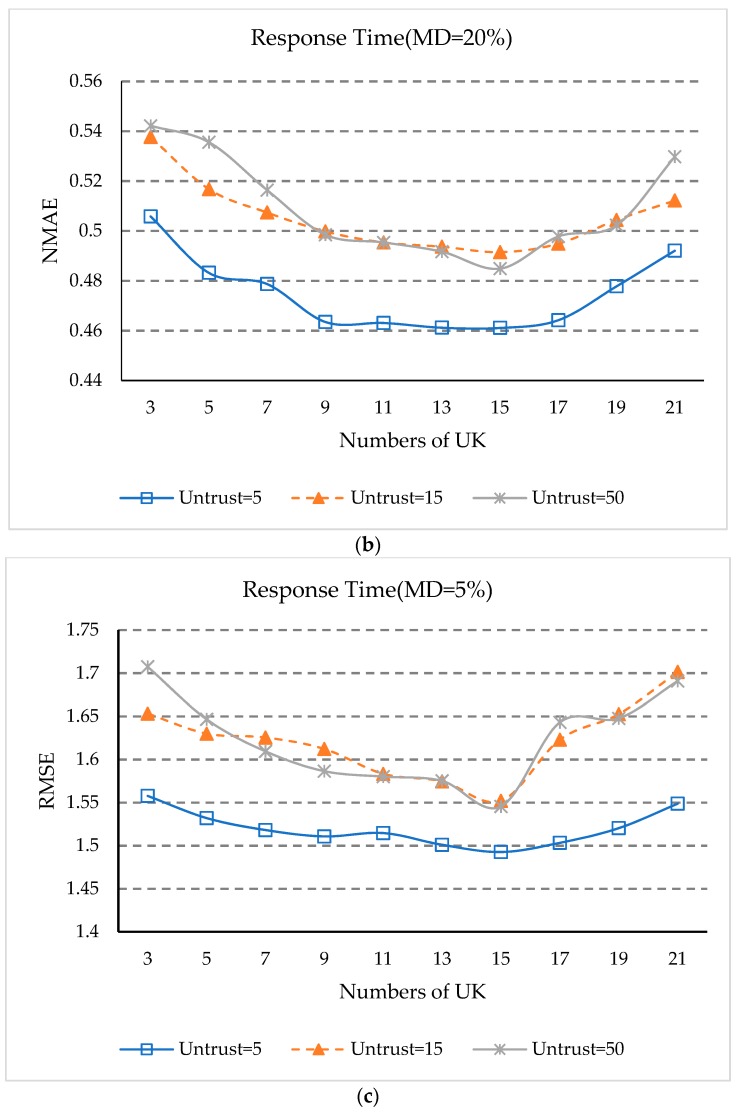

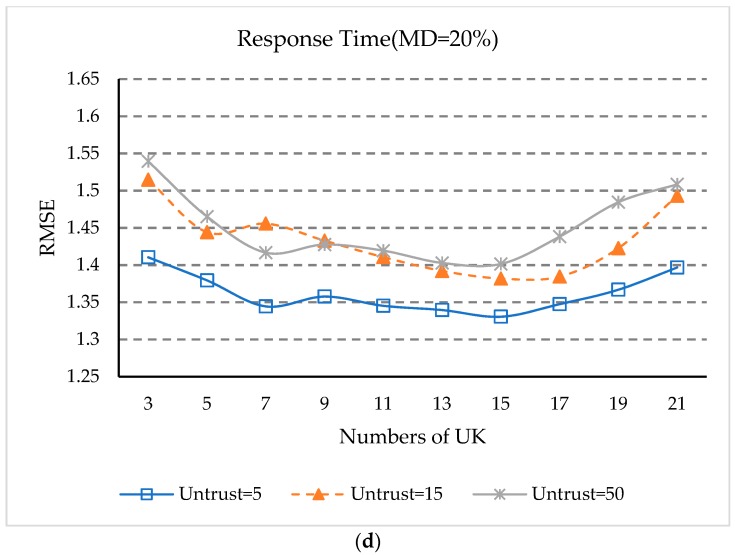
(**a**) Impact of *UK* (MD = 5%, NMAE); (**b**) Impact of *UK* (MD = 20%, NMAE); (**c**) Impact of *UK* (MD = 5%, RMSE); (**d**) Impact of *UK* (MD = 20%, RMSE).

*UK* denotes the number of statistical intervals. In order to study the effect of parameter *UK* on the prediction accuracy, we change the step size from 3 to 21, with 2 steps. The number of untrusted users is set to 5, 15, and 50, and the other parameters *δ*, *K_S_*, *K_NS_*, and *K_NU_* are the same as Section 5.3. This experiment is performed at 5% and 20% matrix densities. The experimental results are shown in Figure 6.

Experimental results show:

(1) It can be seen from Figure 6 that the values of NMAE and RMSE keep declining when the value of *UK* is between 3 and 15. When the value of *UK* is between 15 and 21, the values of MAE and RMSE begin to slowly rise again. This is because when the number of statistical intervals *UK* is relatively small, the reliable interval cannot distinguish the untrustworthy users. When the statistical interval is relatively large, the reliable interval users may not be able to completely include all the trusted users.
(2) It can also be observed in Figure 6 that when the number of untrusted users is 5, the NMAE and RMSE values are smaller than those of 15 and 50, while when the number of untrustworthy users is 15, the NMAE and RMSE values are generally smaller than for 50 users. This fact shows that when there are more untrusted users, this will still affect the accuracy of QoS prediction.
(3) As can be seen from Figure 6, when *UK* equals 15, the NAME and RMSE values are the smallest under different numbers of untrusted users, which indicates that when the statistical interval *UK* is 15, our approach has the best prediction accuracy.

### 5.7. Impacts of Matrix Density

**Figure 7:**
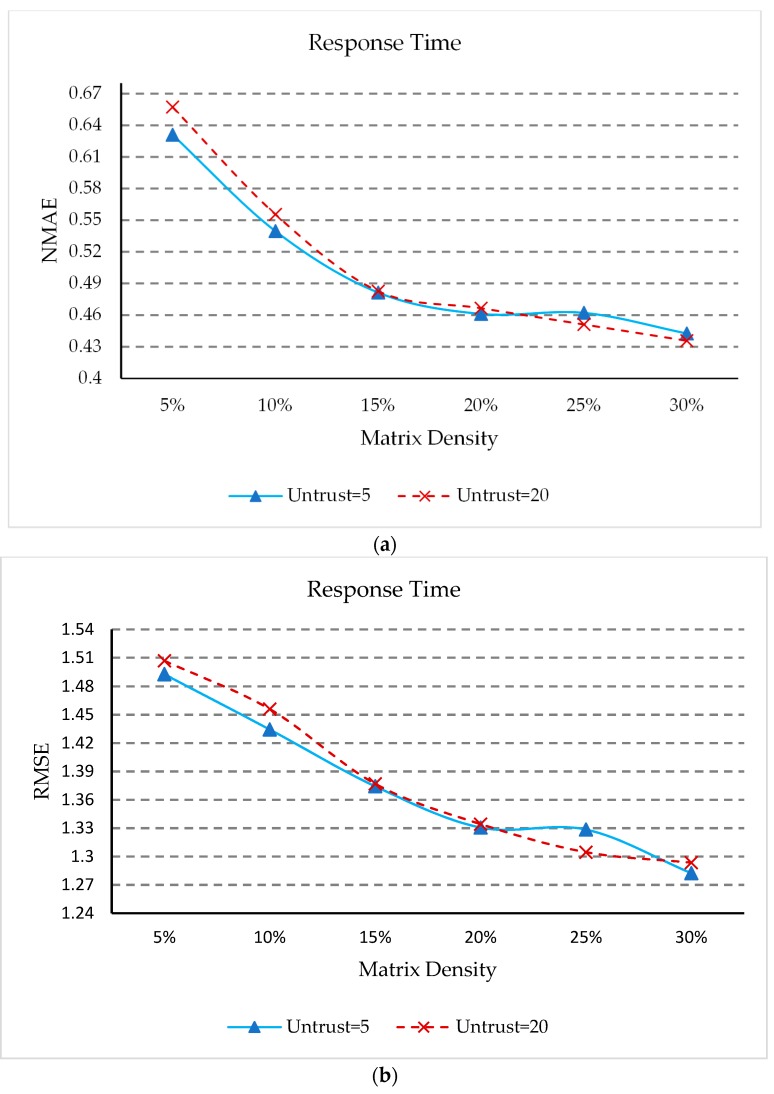
(**a**) Impact of matrix density(NMAE); (**b**) Impact of matrix density(RMSE).

The matrix density is the percentage of entries in the user service QoS matrix which have non-zero values, indicating how many history records in the QoS data can be used to predict the missing entries. In order to study the effect of matrix density on our approach, we changed the matrix density from 5% to 30%, with a step size of 5%. We choose the number of untrusted users to be 5 and 20, and the settings for the other parameters *δ*, *UK*, *K_S_*, *K_NS_*, and *K_NU_* are the same as Section 5.3. The experimental results are presented in Figure 7.

As can be seen from Figure 7, the NMAE and RMSE values decrease rapidly as matrix density increases at first, and then the NMAE and RMSE values decrease at a slower rate when the matrix density becomes larger. This means that as the matrix density increases, more information is available for prediction, so the prediction accuracy will increase; but as the matrix density becomes larger and larger, the focus should be on improving the prediction model. It can also be seen in Figure 7 that in the case of two different untrusted users, the value of NMAE and RMSE are all very close. This shows that our approach can identify untrusted users well, and minimize the impact of untrusted users.

### 5.8. Impact of K_s_

**Figure 8:**
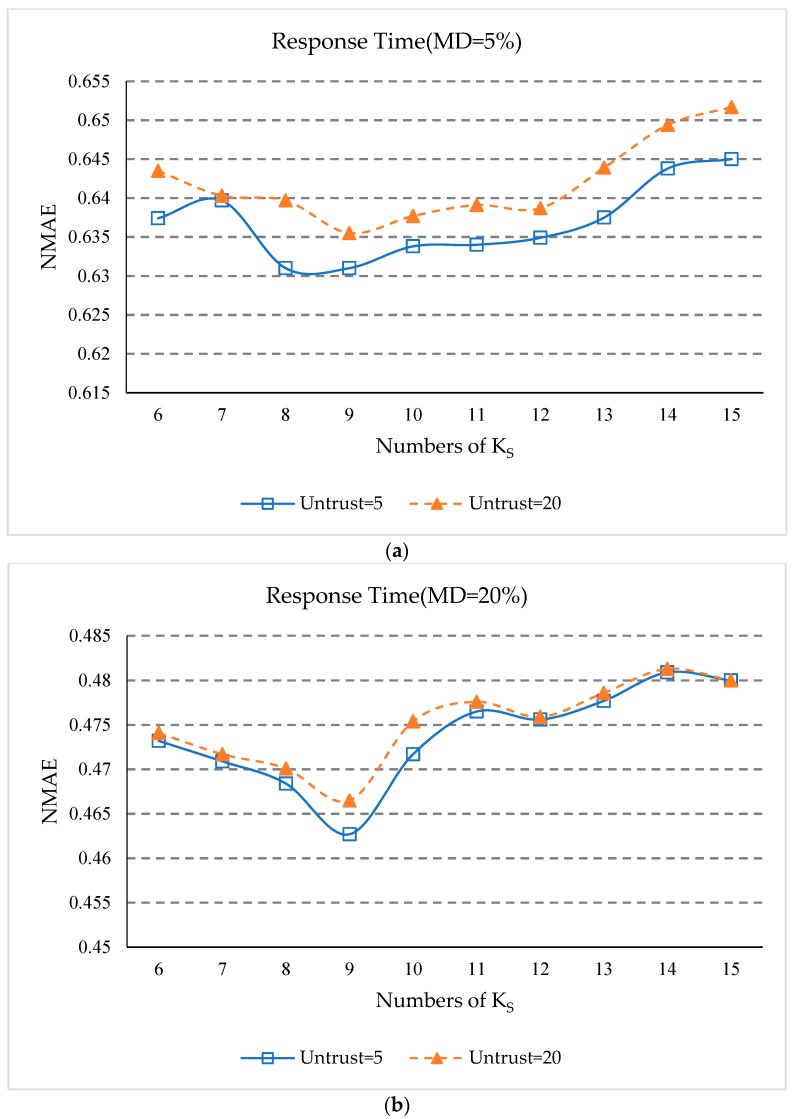

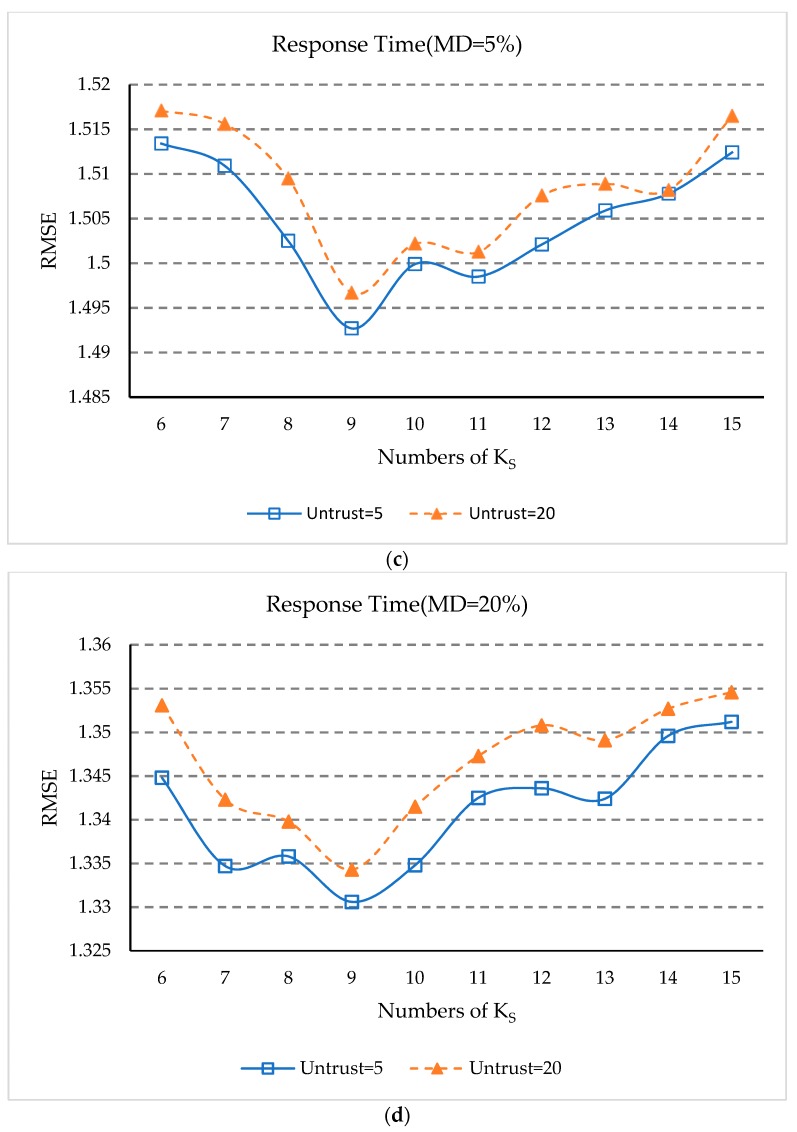
(**a**) Impact of *K_S_* (MD = 5%, NMAE); (**b**) Impact of *K_S_* (MD = 20%, NMAE); (**c**) Impact of *K_S_* (MD = 5%, RMSE); (**d**) Impact of *K_S_* (MD = 20%, RMSE).

In order to study the influence of the number of service clusters *K_S_*, on the prediction accuracy, we do two sets of experiments. The first set of experiments will change *K_S_*, from 6 to 15 with a step size of 1. Other parameters *δ*, *UK*, *K_S_*, *K_NS_*, and *K_NU_* are set the same as in Section 5.3. The matrix density is set to 5% and the number of untrusted users is set to 5 and 20. In another set of experiments, other experimental parameters are set the same as the first set of experiments, except that the matrix density is set to 20%. The experimental results are shown in Figure 8.

Experimental results show:

(1) In Figure 8a,b, the overall trend of NMAE value decreases first and then increases as *K_S_* increases, which indicates that when the number of service clusters is too small, our approach can’t exclude services that are dissimilar but happen to have similar QoS experiences with a few common users. In addition, when the service cluster is too much, the number of services in the service cluster will be too small to find adequate geographic neighbors.
(2) In Figure 8c,d, the overall trend of the RMSE value also decreases first and then increases as *K_S_* increases, but the RMSE value decreases quickly. This is because RMSE evaluation indicators are more susceptible to services that are not truly similar.
(3) As can be seen from Figure 8a–d, when *K_S_* is equal to nine, the values of NMAE and RMSE are the smallest. This shows that when the number of service clusters is nine, our approach has the best prediction accuracy.

### 5.9. Impact of K_NS_ and K_NU_

**Figure 9:**
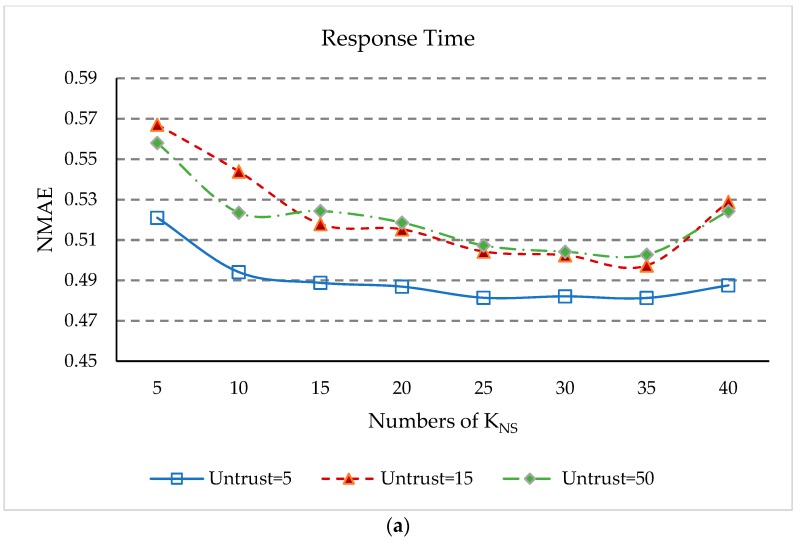

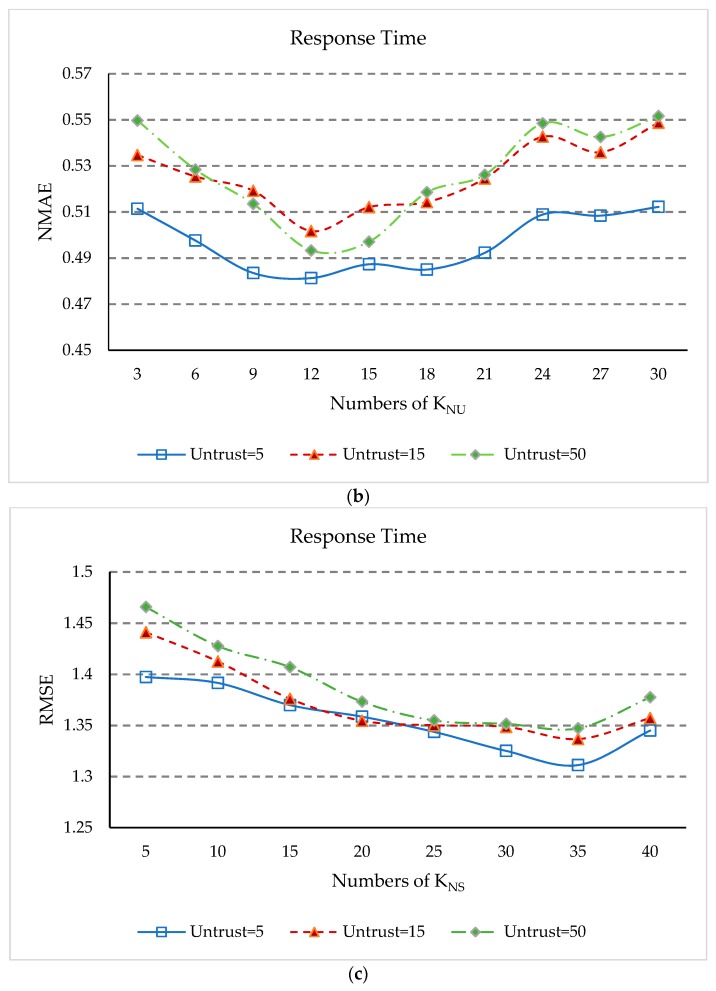

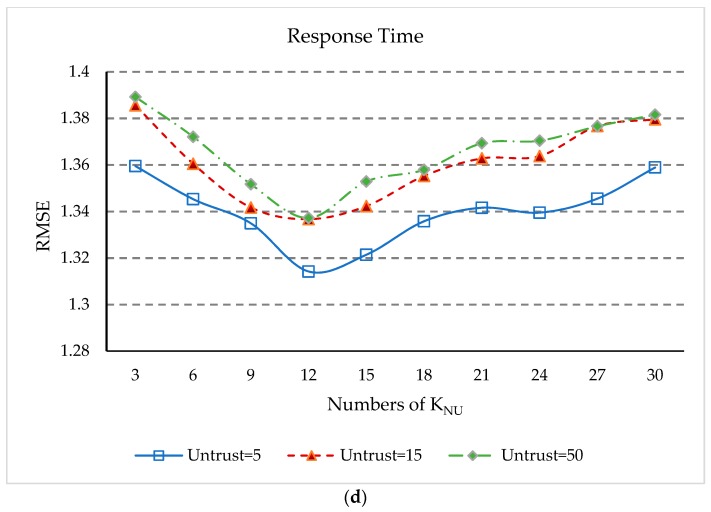
(**a)** Impact of *K_NS_* (NMAE); (**b**) impact of *K_NU_* (NMAE); (**c**) Impact of *K_NS_* (RMSE); (**d**) impact of *K_NU_* (RMSE).

*K_NS_* and *K_NU_* denote, respectively, the number of closest neighbors to a service or user. In order to study the influence of service cluster *K_NS_* and user cluster *K_NU_* on prediction accuracy, we have done two sets of experiments. The first set of experiments will change *K_NS_* from 5 to 40 with a step size of 5. *K_NU_* will be set to 12, other parameters *δ*, *UK*, and *K_S_* are set the same as in Section 5.3. Matrix density is set to 15%, and the number of untrusted users is set to 5, 15, and 50. Another set of experiments will change *K_NU_* from 3 to 30 with a step size of 3. *K_NS_* is set to 35, and the other settings are the same as the first experiment. The experimental results are presented in Figure 9.

Figure 9 shows the effect of the change in parameters *K_NS_* and *K_NU_* on the prediction. We make a couple of observations.

(1) The values of NMAE in Figure 9a and RMSE in Figure 9c initially decrease rapidly with increasing *K_NS_*, but after *K_NS_* is equal to 15, the rate of decline slows down. After *K_NS_* is equal to 35, the values of NMAE and RMSE rose rapidly again. This is because when the number of geographical neighbors of services is too small, our approach cannot obtain enough information to predict unknown QoS values, and when *K_NS_* is too large, the dissimilar geographical neighbors will affect the accuracy of QoS prediction.
(2) When *K_NS_* equals 35, the NMEA value in Figure 9a and the RMSE value in Figure 9c are the smallest in the case of different numbers of untrusted users. This shows that when the service’s similar geographic neighbors equals 35, the QoS prediction accuracy is best.
(3) In Figure 9b,d, the values of NMAE and RMSE begin to decrease. Then, when *K_NU_* equals 12, NMAE and RMSE reach the minimum value. When *K_NU_* is greater than 12, the values of NMAE and RMSE increase continuously. However, when *K_NU_* is greater than 24, the values of NMAE and RMSE increase slowly. The reason is similar to the above, when *K_NU_* increases to a certain level, the impact of this parameter on QoS prediction will be weakened. As can be seen from Figure 9b,d, when *K_NU_* is equal to 12, that is the geographical proximity of the user is 12, the prediction accuracy of our approach is optimal.
(4) Thus, it can conclude that *K_NS_* equal to 35 and *K_NU_* equal to 12 give the best parameter setting for the experimental results in our approach.

## 6. Conclusions and Future Work

User reputation and geographic location information of users and services have significant impacts on QoS prediction for CPS services. In this paper, we propose a QoS prediction approach (GURAP) based on location and reputation-aware collaborative filtering to predict the unknown QoS value for CPS services. The proposed approach includes three algorithms, namely a user reputation calculation algorithm, a user and service similar geographical neighbor identification algorithm, and an unknown QoS prediction algorithm. The user reputation algorithm first calculates the reputation of the user based on the QoS information of the feedback that the user invokes from services and the Dirichlet probability distribution, and then identifies the untrusted user based on the reputation threshold and fixes the unreliable data provided by the untrusted user. The user and service similar geographical neighbor identification algorithm first, according to the collected geographic information of users and services, divides the service and users into three levels of geography level by the method of geographical neighborhood stratification, then improves the PCC formula by the relationship between user and service geographical area. The unknown QoS prediction algorithm predicts unknown QoS values by using similar geographical neighbors of trusted users and services. The three algorithms in our proposed approach can not only identify the untrusted users precisely and mine the nearest geographical neighbors, but also alleviates the problem of matrix sparsity and cold start. Extensive real-world experimental evaluations show that our approach is more accurate and robust than most state-of-the-art methods.

Our GURAP approach uses only the similar geographic neighbor information of users and services to predict the unknown QoS value of trusted users. Since cyber-physical systems can collect the information about the surrounding environment of various devices, we will try to mix objective information of users or services with our methods to improve prediction accuracy, such as the popularity of the service, costs, personal basic information of the user, network conditions of the users or services, and so on. Due to the small number of users of the dataset we use, we cannot cluster users’ latitudes and longitudes, which can affect prediction results. Therefore, in the future, we will collect more real-world datasets to experiment with our QoS prediction framework.

## Figures and Tables

**Table 1:** A toy example of the user-service quality of service (QoS) matrix.

	*S*_1_	*S*_2_	*S*_3_	*S*_4_	*S*_5_
***u*** **_1_**	0.334	0	0.432	0	0
***u*** **_2_**	0	0.938	0	0	1.321
***u*** **_3_**	0.206	0.304	0	0	0
***u*** **_4_**	0	0	0	0.468	0.541

**Table 2:** Prediction accuracy comparison on response time normalized mean absolute error (NMAE). The bold numbers indicate the result of the approach.

Matrices	Methods	Matrix Density(MD)					
		5%	10%	15%	20%	25%	30%
**2.95% Untrustworthy Users**	**UPCC**	0.9229	0.8674	0.8670	0.8451	0.8215	0.7917
	**IPCC**	0.9443	0.8875	0.8706	0.8675	0.8425	0.8014
	**UIPCC**	0.8553	0.7887	0.7863	0.7620	0.7364	0.7206
	**CURA**	0.7745	0.6227	0.5485	0.4882	0.4917	0.4766
	**GUIPCC**	0.8184	0.7650	0.7469	0.7351	0.7094	0.6805
	**TAP**	0.6304	0.5889	0.6043	0.6203	0.6168	0.6070
	**GNMF**	0.6842	0.6471	0.6079	0.5646	0.5465	0.5128
	**GURAP**	**0.6455**	**0.5518**	**0.4887**	**0.4798**	**0.4502**	**0.4354**
**Improvement vs. TAP (%)**		−2.39%	6.30%	19.13%	22.65%	27.01%	28.27%
**Improvement vs. GNMF (%)**		5.66%	14.73%	19.61%	15.02%	17.62%	15.09%
**5.90% Untrustworthy Users**	**UPCC**	0.9369	0.8870	0.8790	0.8467	0.8261	0.7987
	**IPCC**	0.9576	0.8903	0.8755	0.8555	0.8478	0.8159
	**UIPCC**	0.8631	0.8022	0.7892	0.7460	0.7485	0.7325
	**CURA**	0.7883	0.6197	0.5239	0.4891	0.4569	0.4486
	**GUIPCC**	0.8122	0.7809	0.7571	0.7381	0.7190	0.6984
	**TAP**	0.6501	0.5725	0.5991	0.6232	0.6275	0.6258
	**GNMF**	0.6989	0.6265	0.5998	0.5523	0.5264	0.5025
	**GURAP**	**0.6573**	**0.5555**	**0.4826**	**0.4665**	**0.4511**	**0.4357**
**Improvement vs. TAP (%)**		−1.11%	2.97%	19.45%	25.14%	28.11%	30.38%
**Improvement vs. GNMF (%)**		5.95%	11.33%	19.54%	15.53%	14.30%	13.29%

**Table 3:** Prediction accuracy comparison on response time root mean squared error (RMSE). The bold numbers indicate the result of the approach.

Matrices	Methods	Matrix Density(MD)					
		5%	10%	15%	20%	25%	30%
**2.95% Untrustworthy Users**	**UPCC**	2.4882	2.2699	1.9419	1.8698	1.8088	1.7901
	**IPCC**	2.5753	2.3225	2.1973	1.931	1.8888	1.7575
	**UIPCC**	2.3637	2.0274	1.9799	1.8419	1.7403	1.7391
	**CURA**	1.88	1.7196	1.5648	1.5471	1.4329	1.4015
	**GUIPCC**	2.3637	1.974	1.9099	1.8119	1.7003	1.6791
	**TAP**	1.6645	1.6308	1.6446	1.6517	1.6679	1.6851
	**GNMF**	2.2754	2.0785	1.8724	1.7029	1.6823	1.6547
	**GURAP**	**1.4939**	**1.4329**	**1.3757**	**1.3325**	**1.3269**	**1.2845**
**Improvement vs. TAP (%)**		10.25%	12.14%	16.35%	19.33%	20.44%	23.77%
**Improvement vs. GNMF (%)**		34.34%	31.06%	26.53%	21.75%	21.13%	22.37%
**5.90% Untrustworthy Users**	**UPCC**	2.5332	2.3007	2.1174	1.9267	1.8613	1.8157
	**IPCC**	2.6811	2.3964	2.1436	1.9987	1.882	1.8305
	**UIPCC**	2.4767	2.2887	2.0342	1.9264	1.8226	1.793
	**CURA**	1.8871	1.7339	1.5566	1.5582	1.4636	1.4102
	**GUIPCC**	2.4263	2.1584	1.9342	1.8768	1.8023	1.7734
	**TAP**	1.7067	1.6521	1.6563	1.6648	1.6858	1.6904
	**GNMF**	2.333	2.0166	1.8986	1.8069	1.7309	1.6952
	**GURAP**	**1.4967**	**1.4459**	**1.3769**	**1.3343**	**1.3246**	**1.2835**
**Improvement vs. TAP (%)**		12.30%	12.48%	16.87%	19.85%	21.43%	24.07%
**Improvement vs. GNMF (%)**		35.85%	28.30%	27.48%	26.15%	23.47%	24.29%
